# Comparison of adequacy between transbronchial lung cryobiopsy samples and endobronchial ultrasound‐guided transbronchial needle aspiration samples for next‐generation sequencing analysis

**DOI:** 10.1111/1759-7714.13770

**Published:** 2020-12-03

**Authors:** Mari Tone, Minoru Inomata, Nobuyasu Awano, Naoyuki Kuse, Kohei Takada, Jonsu Minami, Yutaka Muto, Kazushi Fujimoto, Toshio Kumasaka, Takehiro Izumo

**Affiliations:** ^1^ Department of Respiratory Medicine Japanese Red Cross Medical Center Shibuya Japan; ^2^ Department of Pathology Japanese Red Cross Medical Center Shibuya Japan

**Keywords:** Bronchoscopy, cryobiopsy, endobronchial ultrasound‐guided transbronchial needle aspiration, lung cancer, next‐generation sequencing

## Abstract

**Background:**

Most lung cancer patients present with lesions in both lung fields and lymphadenopathy. Thus, transbronchial lung cryobiopsy (TBLC) and endobronchial ultrasound‐guided transbronchial needle aspiration (EBUS‐TBNA) are commonly performed for diagnosing lung cancer. However, the adequacy of these samples for next‐generation sequencing (NGS) analysis remains unclear. This study aimed to compare the adequacy between TBLC and EBUS‐TBNA samples for NGS analysis.

**Methods:**

This retrospective cohort study included patients whose lung samples were collected via TBLC or EBUS‐TBNA and analyzed using NGS. Out of 46 genes, the number of genes in TBNA and TBLC samples that could not be assessed via NGS analysis was mainly evaluated.

**Results:**

A total of 37 patients were included and classified into two groups (TBLC group, *n* = 18 and TBNA group, *n* = 19). The mean number of genes that could not be evaluated via NGS analysis was significantly lower in the TBLC group than in the TBNA group (0.9 vs. 10.3, *P* = 0.024). The median total area of tumor cells in TBLC samples was significantly greater than that in TBNA samples (6.3 [1.6–4.2] vs*.* 2.6 [0.2–17.3] mm^2^, *P* < 0.01). In the TBNA group, there were two fully inadequate samples for NGS analysis with a high degree of cell crush or low tumor content, while there was no fully inadequate sample in the TBLC group.

**Conclusions:**

TBLC is more effective in obtaining adequate samples for NGS analysis than EBUS‐TBNA. TBLC should be performed to obtain adequate samples for NGS analysis in lung cancer patients with target lesions in lung fields, even if they have lymphadenopathy.

**Key points:**

**Significant findings of the study**

- The mean number of genes that could not be evaluated was significantly lower in TBLC samples than in EBUS‐TBNA samples (0.9 vs. 10.3, *P* = 0.024). TBLC could obtain adequate samples with a high concentration of uncrushed tumor cells for NGS.

**What this study adds**

- To obtain samples for NGS analysis, the use of TBLC should be aggressively considered in lung‐cancer patients with target lesions located in lung fields, even if they have lymphadenopathy.

## Graphical abstract

The adequacy for next generation sequencing (NGS) in transbronchial lung cryobiopsy (TBLC) and endobronchial ultrasound‐guided transbronchial needle aspiration (EBUS‐TBNA) samples remains unclear. We found the mean number of genes which could not be assessed via NGS was significantly lower in TBLC samples than EBUS‐TBNA samples. TBLC could obtain adequate samples with a high concentration of uncrushed tumor cells for NGS.

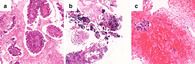

## Introduction

Different molecular targeted drugs are widely used for the treatment of advanced or recurrent non‐small cell lung cancer (NSCLC) harboring driver gene mutations. At present, these drugs are used in clinical practice or ongoing clinical trials on *EGFR* mutations, *ALK* rearrangements, *ROS1* rearrangements, *BRAF V600E* mutation, *MET* exon 14 splice site, and *KRAS G12c* mutation‐positive NSCLC treatment.^1^, ^2^, ^3^, ^4^, ^5^, ^6^, ^7^, ^8^, ^9^, ^10^ In NSCLC harboring driver gene mutations, the effects of molecular targeted drugs are generally better than those of cytotoxic agents. Therefore, NSCLC patients should undergo tests for detecting driver gene mutations before receiving appropriate treatments. Thus far, molecular target tests, such as polymerase chain reaction amplification, immunohistochemistry (IHC), and fluorescence in situ hybridization, are performed to identify a specific driver mutation. However, they require a significant volume of lung samples because each test can only detect one gene information. Next‐generation sequencing (NGS) can be used to screen many types of driver genes in relatively low‐volume samples at once.^11^ In Japan, several NGS systems are utilized as insurance‐coverage tests for lung cancer. The Oncomine Dx Target Test CDx System (SRL, Tokyo, Japan), which is widely used in Japan, can evaluate 46 types of genes at once, while the Oncomine system has been developed as a companion diagnostic test for NSCLC harboring *BRAF V600E*, *EGFR*, *ALK*, and *ROS1*.^12^, ^13^

Previous studies have shown that NGS analysis can be performed using low‐volume tumor samples, such as those obtained via transbronchial lung biopsy (TBLB). However, the success rate of NGS analysis using TBLB samples was relatively low at 15%–82%.^11^, ^14^ In addition, some studies have reported the feasibility of endobronchial ultrasound‐guided transbronchial needle aspiration (EBUS‐TBNA) samples for NGS analysis.^15^, ^16^, ^17^, ^18^ However, we often find that EBUS‐TBNA samples with a high concentration of blood contamination and severely crushed tumor cells cannot be commonly evaluated via NGS analysis in clinical practice. Ideally, tumor samples with a high concentration of tumor cells and without crushed cells should be used in NGS analysis. For instance, the concentration of tumor cells in samples used in the Oncomine system is commonly recommended to be >30%. For obtaining only a pathological diagnosis, an adequate volume of samples can be obtained via EBUS‐TBNA. However, EBUS‐TBNA samples may not be fully adequate for NGS analysis because they can be contaminated with blood or contain crushed tumor cells.

Transbronchial cryobiopsy (TBLC) has emerged as a useful procedure for lung biopsy because it can obtain samples with a larger volume than conventional procedures, such as TBLB.^19^, ^20^ In addition, most tumor cells in samples obtained via TBLC are not crushed, unlike those obtained via EBUS‐TBNA. Either TBLC or EBUS‐TBNA should be considered for lung cancer biopsy because most patients with lung cancer present with lesions in both lung fields as well as lymphadenopathy. However, no study has previously compared the feasibility of using TBLC and EBUS‐TBNA samples for NGS analysis. Herein, the present study was conducted to compare the adequacy between TBLC and EBUS‐TBNA samples for NGS analysis in clinical practice.

## Methods

### Patient selection and data collection

This retrospective cohort study included patients whose lung samples were collected via bronchoscopy and analyzed using the NGS system to detect driver gene mutations at the Japanese Red Cross Medical Center between June 2019 and July 2020. Patients who were diagnosed with primary lung cancer were mainly included, although patients diagnosed with metastatic lung cancer were also included. Patients who received TBLB were excluded in order to compare the adequacy between TBLC and EBUS‐TBNA samples for NGS analysis. TBLC was performed for a peripheral pulmonary target lesion (lobar bronchus) using EBUS or a central pulmonary target lesion (bronchus, lobar bronchi, and segmental bronchi) under direct visualization on bronchoscopy. Meanwhile, TBNA was performed for hilar or mediastinal lymph nodes or a central pulmonary target lesion, which could be approached using a TBNA needle. During bronchoscopy conferences at our institution, whether TBLC or EBUS‐TBNA should be used during a target legion biopsy was discussed in particular when patients had target lesions that could be approached via both TBLC and EBUS‐TBNA. We collected data such as those on the baseline characteristics of patients, computed tomography (CT) scan findings, procedure details, complications, pathological findings, and NGS analysis results from medical charts.

### Procedures

Patients who underwent bronchoscopy received intravenous anesthesia (pethidine and midazolam) and local anesthesia (2% lidocaine intratracheally). During the procedure, the blood pressure, pulse rate, and oxygen saturation of all patients were monitored. To maintain an SpO_2_ of ≥90%, the patients received oxygen therapy while under moderate to deep sedation. All anticoagulant agents were discontinued before the procedure, as per treatment guidelines.^21^ The total procedure duration was defined as time from bronchoscope insertion via the vocal cord until bronchoscope removal from the body. Pneumologists with 2–3 years of bronchoscopy experience performed the procedure.

### TBLC

We performed TBLC with a flexible bronchoscope (EB530T; Fujifilm, Tokyo, Japan) and a 1.9 mm cryoprobe (ERBECRYO 2; Erbe Elektromedizin GmbH, Tubigen, Germany). All patients were intubated with an 8.0 mm endotracheal tube (Suction Above Cuff Endobroncheal Tube, 8.0 mm; Smiths Medical International Ltd., Minneapolis, MN, the USA). The Fogarty catheter (E‐080‐4F; Edwards Life Sciences, Irvine, CA, the USA), which was advanced to the bronchus via the suction channel on the cuff of the endotracheal tube, was routinely used to achieve hemostasis. A 1.4 mm 20‐MHz radical probe (PB2020‐M; Fujifilm, Tokyo, Japan) and a guide sheath (K‐203; Olympus, Tokyo, Japan) were used to identify a target legion in the peripheral pulmonary region and obtain samples at the same location. After identifying the target lesion, the cryoprobe was inserted via the working channel of the bronchoscope and was placed at the desired location under direct visualization on bronchoscopy or X‐ray fluoroscopy. The lesion was frozen for 5–7 seconds using a cryoprobe. The bronchoscope was then immediately removed with the cryoprobe along with the tissues. The Fogarty catheter placed in the segmental or subsegmental bronchus, chosen for TBLC, was inflated for 30–60 seconds immediately after removing the bronchoscope. After the bronchoscope was immediately reinserted to assess for hemostasis, TBLC was repeated until a sample with adequate volume was obtained. The tissue samples were immediately fixed in 10% neutral‐buffered formalin. All procedures were not performed without rapid on‐site cytological evaluation (ROSE).

### EBUS‐TBNA

EBUS‐TBNA was performed with a convex ultrasound bronchoscope (EB530US; Fujifilm, Tokyo, Japan) and a 21‐gauge EBUS needle (NA‐U401SX‐4025N; Olympus, Tokyo, Japan). The target lymph nodes were visualized with EBUS; then, the Doppler blood flow instrument was used to assess blood flow around and within the lymph nodes. Needle passes were provided for 20–30 times during one puncture. After assessing for hemostasis, EBUS‐TBNA was reperformed several times until a sample with adequate volume was obtained. The tissue samples were pushed out from the needle using a stylet. These samples were then immediately fixed in 10% neutral‐buffered formalin. All procedures were not performed without ROSE.

### Sample processing and DNA and RNA sequencing analysis

The tissue samples were fixed in 10% neutral‐buffered formalin for 6–48 hours and embedded in paraffin. Hematoxylin and eosin staining (H&E staining) and IHC staining were performed for histological evaluation, and programmed death‐ligand 1 (PD‐L1) expression was assessed using PD‐L1 IHC 22C3 pharmDx assay (Dako, North America). To obtain a diagnosis, each pathological specimen was evaluated by an experienced pathologist. Moreover, the sample size and total area of tumor cells were counted on H&E‐stained slides using Image J (Image Processing and Analysis in Java, National Institutes of Health, Maryland, USA).^22^ Using formalin‐fixed paraffin‐embedded tissues, 5–10 slides with 5 μm sliced tissues were prepared for NGS analysis using the Oncomine Dx Target Test CDx System. In total, 46 types of gene mutation were detected (Table 1). This analysis method was used in a previous study.^13^ Each type of gene could not be assessed occasionally if the tissue samples were inadequate for NGS analysis; this is referred to as the no call gene. The presence and number of no call genes in each tissue sample in the TBNA and TBLC groups were assessed. Further, the type of gene mutation detected was evaluated.

**Table 1:** Target gene mutation in next‐generation sequencing (NGS) analysis

DNA						
	ALK	AR	BRAF	CTNBB1	DDR2	EGFR
	ERBB2	ERBB3	ERBB4	ESR1	FGFR2	FGFR3
	GNA11	GNAQ	HRAS	IDH1	IDH2	JAK1
	JAK2	JAK3	KIT	KRAS	MAP2K1	MAP2K2
	MET	MTOR	NRAS	PDGFRA	PIK3CA	RAF1
	RET	ROS1	SMO			
RNA						
	ABL1	ALK	AXL	BRAF	ERBB2	ERG
	ETV1	ETV3	ETV5	FGFR1	FGFR2	FGFR3
	MET	NTRK1	NTRK2	NTRK3	PDGFRA	PPARG
	RAF1	RET	ROS‐1			

NGS, next‐generation sequencing.

### Statistical analysis

To evaluate the baseline characteristics of the participants and characteristics of lung specimens detected via NGS analysis, the Fisher's exact test was used to assess categorical data, and the Mann–Whitney U test and *t*‐test were used to evaluate numerical data. The descriptive statistics presented in the current study included means, medians, frequencies, and percentages. All reported *P*‐values were two‐sided, and *P*‐values <0.05 were considered statistically significant. All statistical analyses were performed using EZR (Saitama Medical Center, Jichi Medical University, Saitama, Japan), a graphical user interface for R (the R Foundation for Statistical Computing, Vienna, Austria).

### Ethical considerations

This retrospective study was approved by the institutional review board of the Japanese Red Cross Medical Center (Number 1124) and was registered with the University Hospital Medical Information Network (UMIN000041699). Due to the retrospective nature of the study and based on the Japanese ethical guidelines for clinical research, the need for informed consent was waived.

## Results

### Baseline characteristics of patients

**Figure 1:**
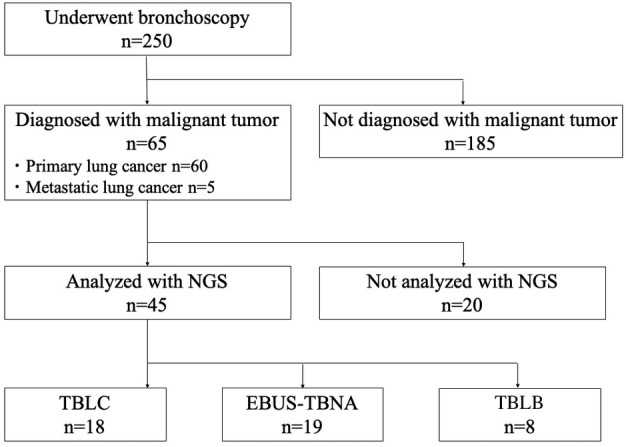
Disposition of patients enrolled in the current study. NGS, next‐generation sequencing; EBUS‐TBNA, endobronchial ultrasound‐guided transbronchial needle aspiration; TBLC, transbronchial lung cryobiopsy; TBLB, transbronchial lung biopsy. [Correction added on 8 December 2020, after first online publication: in figure 1, “TBLC n=8” has been amended to “TBLB n=8”.]

During the study period, 65 of 250 patients who underwent bronchoscopy were diagnosed with a malignant tumor at our institution (primary lung cancer, *n* = 60; metastatic lung cancer, *n* = 5) (Fig 1). A total of 45 patients underwent NGS analysis using bronchoscopy samples to detect driver gene mutations, while NGS analysis was not performed in the remaining 20 patients based on the judgment of the attending physicians. The lung samples were collected via TBLC in 18 patients and EBUS‐TBNA in 19 patients, while eight patients whose lung samples were collected via TBLB were excluded. In total, 37 patients were included in the current study. There was no significant difference in patient baseline characteristics between the TBLC and TBNA groups (Table 2). A total of 11 (61.1%) patients in the TBLC group had lymphadenopathy which could be approached via EBUS‐TBNA, and seven (36.8%) in the TBNA group had lesions in lung field, which could be approached via TBLC. Except for two patients in the TBLC group, EBUS was performed to detect target lesions during the procedures in 35 patients. The median frequency of TBLC was one (range: 1–6) in the TBLC group, and that of needle punctures was four (range: 1–6) in the TBNA group. No severe‐grade complication was observed.

**Table 2:** Baseline characteristics of patients at the time of bronchoscopy

Variables	All patients (*n* = 37)	TBLC group (*n* = 18)	TBNA group (*n* = 19)	*P‐*value
Age, years	70 (47–85)	70 (47–82)	71 (56–85)	0.43
Male/female	27 (73.0)/10 (27.0)	13 (72.2)/5 (27.8)	14 (73.7)/5 (26.3)	1.0
ECOG PS score of 0–1/2	34 (91.9)/3 (8.1)	17 (94.4)/1 (5.6)	17 (89.5)/2 (10.5)	1.0
Smoker	32 (86.5)	16 (88.9)	16 (84.2)	1.0
Brinkman index	850 (0–2120)	820 (0–1650)	860 (0–2120)	0.58
Location of target legion				<0.01
Peripheral pulmonary lesions (lobar bronchus)	14 (37.8)	14 (77.8)	0 (0)	
Central pulmonary lesions (bronchus, lobar bronchi, segmental bronchi)	7 (18.9)	4 (22.2)	3 (15.8)	
Mediastinal lymph nodes	14 (37.8)	0 (0)	14 (73.7)	
Hilar lymph nodes	8 (21.6)	0 (0)	8 (42.1)	
Maximum diameter of the target pulmonary lesion or lymph nodes on chest CT scan, mm	38 (8–92)	45 (7–92)	32 (8–68)	0.086
Clinical stage at the time of bronchoscopy				0.61
I	2 (5.4)	2 (11.1)	0 (0)	
II	4 (10.8)	3 (16.7)	1 (5.4)	
III	8 (21.6)	4 (22.2)	9 (47.3)	
IV	18 (48.6)	9 (50.0)	9 (47.3)	
Duration of procedure, minute	29 (15–72)	32 (19–72)	26 (15–55)	0.15

Data are presented as n (%) or median (range).

CT, computed tomography; ECOG PS, Eastern Cooperative Oncology group performance status; TBLC, transbronchial lung cryobiopsy; TBNA, transbronchial needle aspiration.

### Specimens

**Figure 2:**
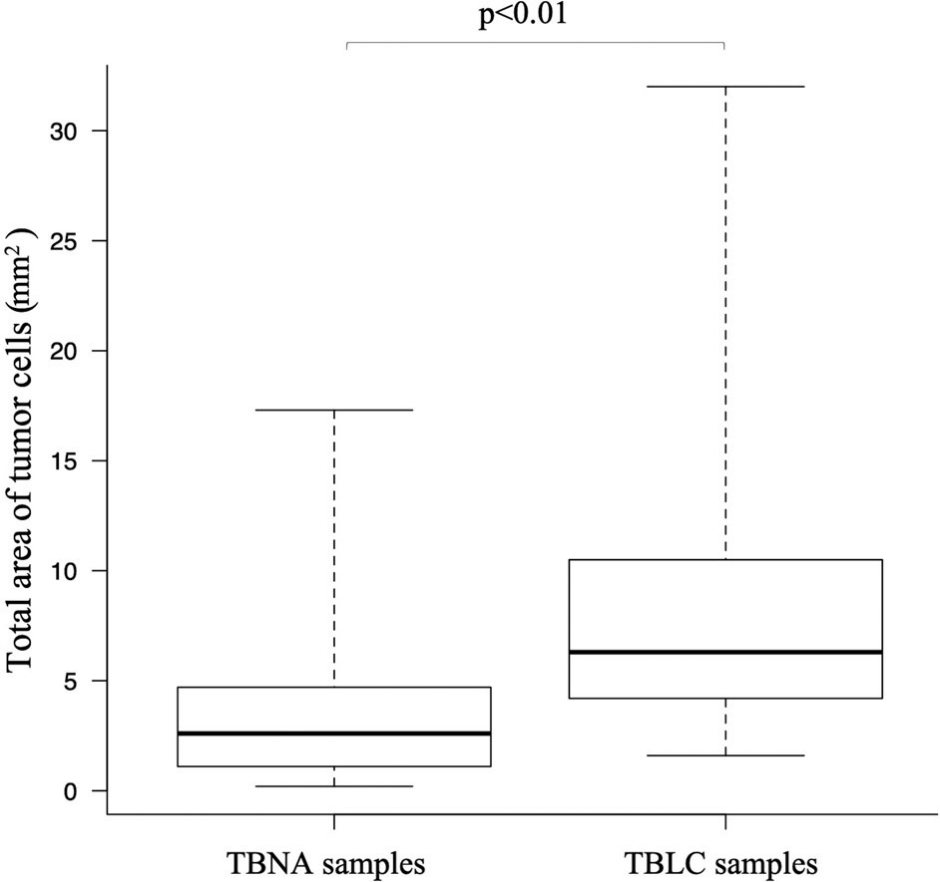
Comparison of the median total area of tumor cells between EBUS‐TBNA and TBLC (2.6 [0.2–17.3] vs. 6.3 [1.6–4.2] mm^2^). EBUS‐TBNA, endobronchial ultrasound‐guided transbronchial needle aspiration; TBLC, transbronchial lung cryobiopsy.

The median total area of samples in the TBLC group was significantly smaller than that in the TBNA group (8.5 [3.6–32.0] vs. 20.0 [0.8–79.7] mm^2^, *P* < 0.01) (Table 3). However, the median total area of tumor cells was significantly greater in the TBLC samples than that in the TBNA samples (6.3 [1.6–4.2] vs. 2.6 [0.2–17.3] mm^2^, *P* < 0.01) (Fig 2). Tumor content proportion in the TBLC samples was also significantly higher than that in the TBNA samples (80.0 [13.1–100] vs. 12.2 [0.5–56.7] %, *P* < 0.01).

**Table 3:** Pathological findings of bronchoscopy specimens

Variables	All patients (*n* = 37)	TBLC group (*n* = 18)	TBNA group (*n* = 19)	*P‐*value
Area of the samples, mm^2^	14.7 (0.8–79.7)	8.5 (3.6–32.0)	20.0 (0.8–79.7)	<0.01
Area of tumor cells in the samples, mm^2^	4.2 (0.2–17.3)	6.3 (1.6–4.2)	2.6 (0.2–17.3)	<0.01
Tumor content, %	44.9 (0.50–100)	80.0 (13.1–100)	12.2 (0.50–56.7)	<0.01
Pathological findings				0.17
Lung squamous cell carcinoma	18 (48.6)	6 (33.3)	11 (57.9)	
Lung nonsquamous cell carcinoma	17 (45.9)	10 (55.6)	8 (42.1)	
Others	2 (5.5)	2 (11.1)	0 (0)	
PD‐L1				0.59
0%	10 (27.1)	5 (27.8)	5 (26.3)	
1%–49%	12 (32.4)	7 (38.8)	5 (26.3)	
50%–	14 (37.8)	5 (27.8)	9 (47.4)	
Unknown	1 (2.7)	1 (5.6)	0 (0)	

Data are presented as n (%) or median (range).

PD‐L1, programmed cell death 1‐ligand 1; TBLC, transbronchial lung cryobiopsy; TBNA, transbronchial needle aspiration.

### Diagnoses

Pathological diagnosis of lung samples was lung squamous cell carcinoma in six patients (33.3%), lung nonsquamous cell carcinoma in 11 patients (66.2%), and metastatic adenocarcinoma in one patient (5.5%) in the TBLC group, while it was lung squamous cell carcinoma in 11 patients (57.9%) and lung nonsquamous cell carcinoma in eight patients (42.1%) in the TBNA group (Table 3). PD‐L1 expression was evaluated, except for one patient in TBLC group. All specimens were adequate for PD‐L1 expression analysis. There was no significant difference in PD‐L1 expression in both groups (Table 3).

### DNA and RNA sequencing analysis

**Figure 3:**
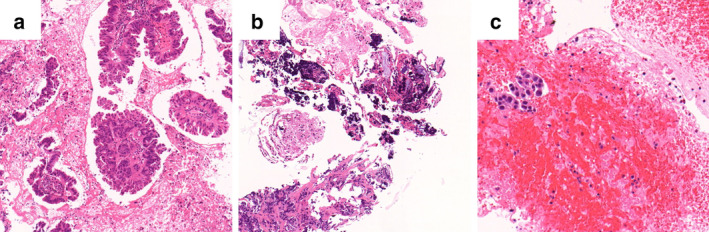
Pathological findings of bronchoscopy in representative cases. (**a**) A fully adequate TBLC sample for NGS analysis. Adequate number of uncrushed tumor cells were observed. (**b**) A fully inadequate EBUS‐TBNA sample for NGS analysis. A high degree of crushed tumor cells was detected. (**c**) A fully inadequate EBUS‐TBNA sample for NGS analysis. There was a large volume of contaminated blood, and the sample had few tumor cells. TBLC, transbronchial lung cryobiopsy; NGS, next‐generation sequencing; EBUS‐TBNA, endobronchial ultrasound‐guided transbronchial needle aspiration.

In the TBLC group, there were 12 fully adequate samples (66.7%; without no call gene) and six partly inadequate specimens (33.3%; with several no call genes) for NGS analysis. Meanwhile, there was no fully inadequate samples (0%; with 46 no call genes) for NGS analysis (Table 4). By contrast, there were two (10.6%) fully inadequate samples, seven (36.8%) fully adequate samples, and 10 (52.6%) partly inadequate samples in the TBNA group. The TBLC samples were more adequate for NGS analysis than the TBNA samples (*P* = 0.11). A high degree of cell crush or low tumor content was observed in two fully inadequate samples for NGS analysis in the TBNA group (Fig 3). Of the 46 types of genes evaluated via NGS analysis, the mean number of no call genes was significantly lower in the TBLC samples than in the EBUS‐TBNA samples (0.9 vs. 10.3, *P* = 0.024). In addition, the TBLC group had a higher number of driver gene mutations than the TBNA group (66.7% vs. 26.3%, *P* = 0.022). The following driver gene mutations were detected via NGS analysis: *EGFR* 5, *KRAS* 3, *ERBB* 2, *MET* 1, and P*IK3CA* 1 in the TBLC group and *EGFR* 1, *KRAS* 1, *MET* 1, *ROS1* 1, and *PIK3CA* 1 in the TBNA group.

**Table 4:** Results of NGS using bronchoscopy samples

	All patients (*n* = 37)	TBLC group (*n* = 18)	TBNA group (*n* = 19)	*P*‐value
Adequate samples for NGS analysis				0.11
Fully adequate samples	19 (51.4)	12 (66.7)	7 (36.8)	
Partly inadequate samples	16 (43.2)	6 (33.3)	10 (52.6)	
Fully inadequate samples	2 (5.4)	0 (0)	2 (10.6)	
Mean number of no call genes	5.7 (0–46)	0.9 (0–10)	10.3 (0–46)	0.024
Driver gene mutation detected via NGS analysis	15 (40.5)	12 (66.7)	5 (26.3)	0.022

Data are presented as n (%) or mean (range).

NGS, next‐generation sequencing; TBNA, transbronchial needle aspiration; TBLC, transbronchial lung cryobiopsy.

## Discussion

The current retrospective cohort study revealed that TBLC samples were superior to EBUS‐TBNA samples for NGS analysis in terms of adequacy. The median total area of tumor cells in the TBLC samples was significantly greater than that in the TBNA samples. In addition, of the 46 genes evaluated via NGS analysis, the mean number of genes that could not be evaluated was significantly lower in the TBLC samples than in the EBUS‐TBNA samples.

In this study, TBLC was more likely to obtain fully adequate samples for NGS analysis than EBUS‐TBNA. Previous studies have reported the use of sample collection methods, which include pathological findings, molecular target tests, and NGS analysis, via EBUS‐TBNA for diagnosing lung cancer.^15^, ^16^, ^17^, ^18^, ^23^, ^24^, ^25^, ^26^ In these studies, about 82.8%–95.3% of EBUS‐TBNA samples were successfully utilized in NGS analysis.^15^, ^16^, ^17^ The current study obtained a similar result showing that the success rate of NGS analysis was 89.4% (fully adequate samples, 36.8%; partly adequate samples, 52.6%). TBLC is a relatively novel procedure for obtaining lung tissue samples. Several studies have used this procedure for the diagnosis of lung cancer along with pathological and molecular target testing.^14^, ^19^, ^20^, ^27^, ^28^, ^29^, ^30^ The success rate of TBLC samples for NGS analysis was high at 100% in the current study (fully adequate samples, 66.7%; partly adequate samples, 33.3%), and this result is similar to those of previous studies (90%–100%).^14^, ^27^

The current study showed that the median total area of tumor cells in TBLC samples was significantly greater than that in TBNA samples while the median area of the TBLC samples was significantly smaller than that of the EBUS‐TBNA samples. Low tumor content in the EBUS‐TBNA samples was probably associated with the failure of NGS analysis. Moreover, there was a high concentration of crushed tumor cells and low tumor content in fully inadequate samples for NGS analysis in the TBNA group. Blood contamination was also detected in the EBUS‐TBNA samples, as reported in previous studies.^31^, ^32^ These unfavorable conditions detected on the EBUS‐TBNA samples could inhibit NGS analysis. Because NGS analysis requires a definite amount of DNA and RNA, a sample with a high number of uncrushed tumor cells should be used. In several studies, the TBLC samples have previously been reported to have a high number of tumor cells and concentration of high‐quality DNA and RNA.^14^, ^27^, ^30^ Thus, with the use of TBLC samples, we are able to obtain samples with not only adequate volumes but also good conditions for NGS analysis, such as those with low concertation of blood contamination or with uncrushed tumor cells.

The current study showed that the TBLC group had a higher number of driver gene mutations than the TBNA group. This result might be influenced by not only differences in pathological features but also those in biopsy procedures between the two groups. The most beneficial biopsy procedure should be considered because the treatment and prognosis of lung cancer are significantly dependent on whether lung‐cancer patients have driver gene mutations.

Most patients diagnosed with advanced lung cancer present with lesions in their lung fields and lymph nodes. In the current study, approximately 50% of patients had both pulmonary lesions, which could be approached via TBLC, and lymphadenopathy, which could be approached via EBUS‐TBNA. EBUS‐TBNA is now widely used and is aggressively performed in clinical practice because sample collection via this procedure is extremely useful for pathological diagnosis and safety. The choice between TBLC and EBUS‐TBNA may depend on the place and size of target lesions. However, considering our study results, TBLC should be more aggressively chosen over EBUS‐TBNA with consideration of NGS analysis. Previous studies have confirmed the safety of TBLC, as in the current study.^14^, ^19^, ^20^, ^29^ However, TBLC can only be performed in limited institutions because of the following reasons. First, it is generally time‐consuming for physicians to train and acquire the TBLC technique. Second, the initial cost of introducing TBLC is high. Hereafter, at many institutions, bronchoscopists should consider the use of TBLC for lung biopsy as it is an effective and safe procedure for the diagnosis of lung cancer, including NGS analysis.

The current study had several limitations. A small sample size was included, and in addition, this was a retrospective study performed at a single institution. Because TBLC and EBUS‐TBNA were not performed on the same patient, the difference in patient characteristics including pathological findings may to some extent influence the results of the study. The current study recommends the use of TBLC in obtaining lung samples for NGS analysis. However, some patients do not have target legions that can be approached via TBLC, and as such they must undergo other procedures other than TBLC for biopsy. In addition, the concentration of DNA and RNA could not be evaluated because this study was performed in clinical practice at a community hospital. Hence, if possible, a large‐scale prospective cohort study should be conducted to further elucidate the difference between TBLC and EBUS‐TBNA samples for NGS analysis in terms of adequacy in each patient.

In conclusion, more adequate samples with a high concentration of uncrushed tumor cells for NGS analysis can be collected with TBLC compared with EBUS‐TBNA. To provide appropriate treatment based on the NGS analysis findings, the use of TBLC should be considered in lung‐cancer patients with target lesions located in lung fields even if they have lymphadenopathy.

## Disclosure

This research did not receive any specific grants from funding agencies in the public, commercial, or not‐for‐profit sectors.
